# Application of 2D Gait Analysis for the Assessment of Gait Disturbance in Patients with Spastic Tetraparesis

**DOI:** 10.17691/stm2021.13.5.03

**Published:** 2021-10-29

**Authors:** A.S. Eliseev, S.Ya. Kalinina, K.S. Yashin, A.S. Zolotova, I.N. Morozov, K.V. Slavin

**Affiliations:** Assistant, Kolokoltsev Department of Traumatology, Orthopedics and Neurosurgery Privolzhsky Research Medical University, 10/1 Minin and Pozharsky Square, Nizhny Novgorod, 603005, Russia; Assistant, Kolokoltsev Department of Traumatology, Orthopedics and Neurosurgery Privolzhsky Research Medical University, 10/1 Minin and Pozharsky Square, Nizhny Novgorod, 603005, Russia; Assistant, Kolokoltsev Department of Traumatology, Orthopedics and Neurosurgery Privolzhsky Research Medical University, 10/1 Minin and Pozharsky Square, Nizhny Novgorod, 603005, Russia; Student Privolzhsky Research Medical University, 10/1 Minin and Pozharsky Square, Nizhny Novgorod, 603005, Russia; Head of the Center for Neurorehabilitation in Spine and Spinal Cord Disorders Privolzhsky Research Medical University, 10/1 Minin and Pozharsky Square, Nizhny Novgorod, 603005, Russia; Professor, Head of the Stereotaxic and Functional Neurosurgery Department University of Illinois at Chicago, 1200 W Harrison St., Chicago, IL, 60607, USA

**Keywords:** 2D gait analysis, gait in spastic tetraparesis, spastic tetraparesis, gait biomechanics in tetraparesis

## Abstract

**The aim of the study** was to explore the use of 2D gait analysis for assessing gait abnormalities in patients with spastic tetraparesis associated with spinal cord injury and other lesions of the cervical spinal cord.

**Materials and Methods:**

The study included 12 patients with tetraparesis of various etiologies. Gait assessment was performed by video analysis using reflective markers (1.5 cm) and a special walking platform. The spatial coordinates of the markers were determined by capturing the reflected light with infrared LEDs located around the lenses of video cameras.

**Results:**

Using 2D gait analysis, numerical indicators of gait disturbance in spastic tetraparesis were obtained. We found a prolongation of the stand phase with a shortening of the swing phase (from 81.9 [76.1; 89.2] to 85.3 [74.4; 90.2]%; p=0.97) and the period of the double step (from 0.50 [0.45; 0.96] to 0.40 [0.34; 0.66]; p=0.4) in comparison with the target (normal) values (60% — for the stand phase; 1.41 — for the double-step period). The movements in the hip, knee, and ankle joints are described using numerical values.

We then compared the data obtained from the left and right sides of the patient’s body: there were no statistically significant differences between the two sets of data. We also compared the gait characteristics before and after treatment (in 4 patients). Statistically significant differences in values were obtained for the stand and swing phases (p=0.035), the range of motion in the hip joint (p=0.01), and gait velocity (p=0.046). Kendall’s analysis revealed no significant correlation between the data obtained by video gait analysis and the gait changes by the Modified Ashworth Scale (р>0.05).

**Conclusion:**

2D gait analysis is a promising method for quantifying gait disturbance in patients with spastic tetraparesis. It allows one to identify characteristic gait patterns, in particular, an increase in the stand phase with a shortening of the swing phase and the double step period, as well as a decrease in the range of motion in the hip joints with an increase in the knee and ankle ones.

## Introduction

An increase in muscle tone of the upper and lower limbs is symptomatic for many diseases affecting the cervical spine. Up to 93% of patients with spinal cord trauma at the cervical level and up to two-thirds of patients with spondylogenic and vascular myelopathy show signs of muscle spasticity [1, 2]. In most cases, an increase in tone leads to gait abnormalities and, as a consequence, to a low quality of life and a high percentage of disability in this group of patients [3–5].

Currently, conservative and surgical treatments are being used either as monotherapy or in combination. However, assessing the treatment effectiveness remains relevant, especially in clinical practice, where subjective judgments about gait disturbance severity are common.

The literature describes clinical, biomechanical, and electrophysiological methods for evaluating the effectiveness of treatment in patients with increased tone of the upper and lower limbs [6]. For this purpose, the Modified Ashworth Scale is most often used in clinical practice [7, 8]. Using this scale, an investigator obtains a subjective assessment of muscle tone. Biomechanical measurements with isokinetic dynamometers were attempted, which made it possible to quantify spasticity; however, this method is rarely used in everyday clinical practice. Electromyography (an electrophysiological method) is also used to measure the muscle tone, but this technique is neither simple nor reliable for the assessment of spasticity [6].

There are recent publications showing the possibility of using a video-based 2D gait analysis (2DGA) — to diagnose spastic tetraparesis and evaluate the effectiveness of its treatment; however, the results pertained to patients with cerebral palsy only [9].

The authors of the present study have obtained their first results of using 2DGA to assess gait disorders in patients with spastic tetraparesis associated with spinal cord injury and other diseases. These results confirm that using this technology makes it possible to quantitatively assess the state of the patient’s gait and choose an effective method of treatment.

**The aim of this study** was to explore the use of 2D gait analysis for assessing gait abnormalities in patients with spastic tetraparesis associated with spinal cord injury and other lesions of the cervical spinal cord.

## Materials and Methods

### Patients

The study included 12 patients (10 men, 2 women) with tetraparesis of various etiologies and the mean disease duration of 5.0 [1.9; 8.5] years.

The work was carried out in accordance with the Helsinki Declaration (2013) and approved by the Ethics Committee of the Privolzhsky Research Medical University (Nizhny Novgorod, Russia). Each patient signed an informed consent to participate in the study.

In 7 cases, spastic tetraparesis resulted from spinal cord injury, in 2 cases — from spondylogenic myelopathy associated with degenerative-dystrophic spinal changes, and in 3 cases — from vascular myelopathy due to spinal stroke. In all patients included in the study, the topical lesion was located in the cervical spinal cord below the C_2_ level.

In 5 cases, the treatment included the implantation of an intrathecal baclofen pump, in 4 cases — the implantation of epidural electrodes, and in 3 cases, conservative therapy was used (medications that improve blood circulation and neuromuscular conduction, physiotherapy, massage, rehabilitation exercises). Hypertonicity was assessed using the Modified Ashworth Scale.

### 2D gait analysis

Gait disturbances were assessed by video analysis with the help of passive markers (1.5 cm) containing reflective material and a special walking platform. The spatial coordinates of the markers were determined by capturing the reflected light with infrared LEDs around the lenses of video cameras [10].

**Figure 1.**
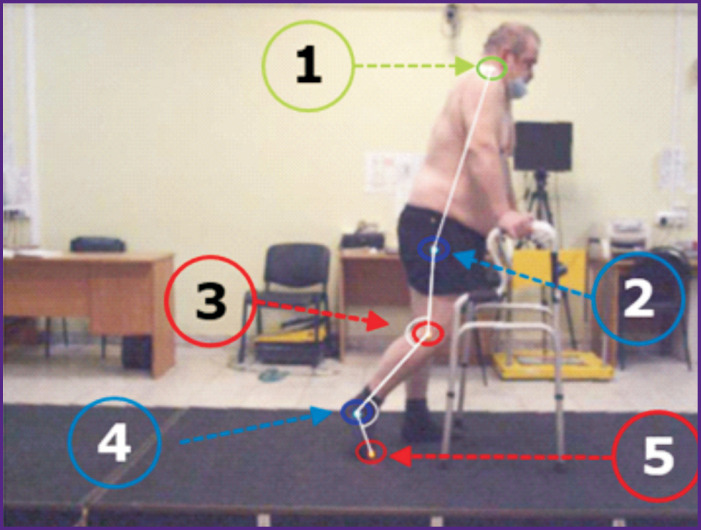
Location of reflective sensors on the patient’s body in the projection of the joints: *1* — shoulder joint, the acromion area; *2* — hip joint, the femoral head area; *3* — knee joint, the projection of the lateral tibial condyle; *4* — ankle joint, the projection of the lateral ankle; *5* — the head of the V metatarsal bone

According to the sagittal gait analysis protocol, 5 markers were attached to the skin at the key anatomical points as suggested by the Simi Reality Motion Systems 2D program (Germany) (Figure 1). Patients moved using a walker (width — 54.5 cm, length — 47.5 cm, the height was adjusted individually).

**Figure 2.**
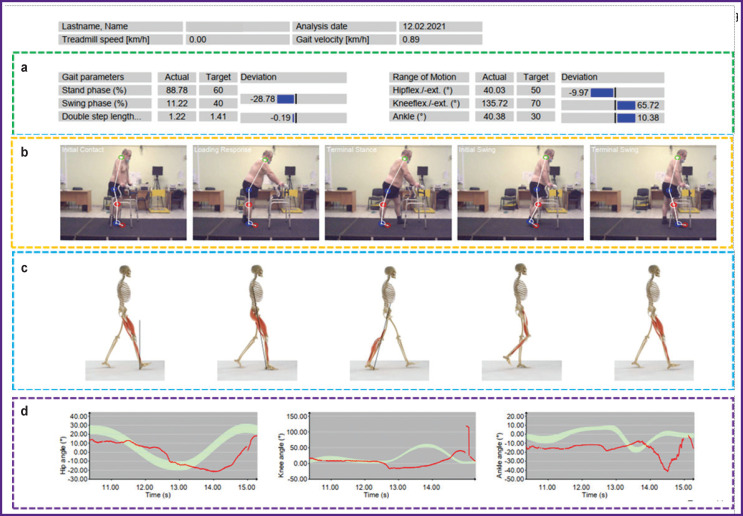
Numerical (a), visual (b), simulated (c), and graphical (d) representations of the kinematic characteristics of walking based on the data comparison between the patient’s real-time movement and the normal gait model

For two-dimensional gait analysis, video images are recorded using a double-axis coordinate system, which requires the data to be recorded separately from the right and left sides of the patient’s body. After the gait test, a report is generated based on the patient’s movement data in real-time with a visual representation of the kinematic characteristics (Figure 2).

During the study, the following gait parameters were assessed: stand phase, swing phase, double step length, range of motion during hip extension (hipflex), knee flexion (kneeflex), and ankle joint flexion. Gait velocity was also analyzed. For each of the parameters, the actual results and their deviation from to the target values were assessed. The target values reflecting the normal gait characteristics were obtained at the University Clinic of Privolzhsky Research Medical University earlier at the stage of setting up the program [11].

### Statistical analysis

Statistical processing was performed using MS Excel 2010 and Statistica 10.0 software. The Kolmogorov–Smirnov analysis showed the absence of a normal distribution in the sample (p<0.05). Differences between groups were assessed using the Mann–Whitney test. The median and interquartile range were used to express the numerical data. Differences were considered statistically significant at p<0.05.

## Results

### Comparison of 2DGA gait parameters obtained from the right and left sides of the patient’s body

Comparative data evaluation produced no statistically significant difference in values between the left and right sides of the body, before treatment (Table 1). Notably, the target (normal) values were not seen in any patient.

**Table 1:** The gait parameters on both sides of the patients’ body before treatment, obtained by 2D gait analysis, Me [Q1; Q3]

Parameter	Target value	Right side	Left side	p
***Changes at the moment***				
Gait parameters:				
stand phase (%)	60.0	81.9 [76.1; 89.2]	85.3 [74.4; 90.2]	0.97
swing phase (%)	40.0	18.0 [10.7; 23.8]	14.6 [9.7; 25.5]	0.97
double step period	1.41	0.50 [0.45; 0.96]	0.40 [0.34; 0.66]	0.4
Range of motion (degrees):				
hip joint (extension)	40.0	25.5 [21.6; 36.4]	27.9 [22.3; 40.6]	0.67
knee joint (flexion)	135.7	30.9 [24.3; 51.2]	58.8 [34.5; 123.2]	0.08
ankle joint	40.4	19.5 [16.8; 26.3]	22.5 [17.2; 28.6]	0.71
***Deviation***				
Gait parameters:				
stand and swing phase (%)	60.0	–21.9 [–29.2; –16.1]	–25.3 [–30.2; –14.4]	0.97
double step period	1.41	–0.91 [–0.96; –0.45]	–0.98 [–1.0; –0.75]	0.4
Range of motion (degrees):				
hip joint (extension)	40.0	–24.4 [–28.3; –13.5]	–22.1 [–27.6; –0.5]	0.55
knee joint (flexion)	135.7	–39.0 [–45.6; –18.7]	–17.6 [–37.3; 50.0]	0.14
ankle joint	40.4	–10.4 [–13.1; –3.6]	–6.4 [–12.7; 10.6]	0.55
Gait velocity (km/h)	4.50	0.51 [0.26; 1.12]	0.41 [0.25; 1.18]	0.71

### Comparison of 2DGA gait parameters before and after treatment

This comparison was made in 4 patients. The gait improvement was determined according to the following parameters: 1) stand phase and swing phase, 2) range of hip motion, and 3) gait velocity (Table 2). Statistically significant changes were found in the duration of the stand and swing phases (p=0.035), the range of hip motion (p=0.01), and the gait velocity (p=0.046).

**Table 2:** Comparison of gait parameters before and after treatment, Me [Q1; Q3]

Parameter	Before treatment	After treatment	p
***Changes at the moment***				
Gait parameters:				
stand phase (%)	**86.0 [79.3; 90.2]**	**79.5 [72.9; 85.3]**	**0.035**
swing phase (%)	**14.0 [9.7; 20.6]**	**20.4 [14.6; 27.0]**	**0.035**
double step period	0.50 [0.34; 0.96]	0.71 [0.44; 0.99]	0.34
Range of motion (degrees):				
hip joint (extension)	**24.3 [20.0; 29.1]**	**33.4 [24.5; 37.9]**	**0.01**
knee joint (extension)	37.3 [26.0; 80.4]	42.1 [32.5; 55.8]	0.77
ankle joint	20.7 [16.6; 41.6]	39.5 [14.6; 51.3]	0.32
***Deviation***				
Gait parameters:				
stand and swing phase (%)	**–26.0 [–30.2; –19.3]**	**–19.5 [–25.3; –12.9]**	**0.035**
double step period	–0.91 [–1.0; –0.45]	–0.71 [–0.97; –0.42]	0.34
Range of motion (degrees):				
hip joint (extension)	**–25.6 [–29.9; –20.9]**	**–16.5 [–25.4; –11.6]**	**0.011**
knee joint (extension)	–32.6 [–43.9; 10.4]	–27.8 [–37.4; 13.5]	0.67
ankle joint	–9.2 [–13.3; 11.9]	9.5 [–15.3; 31.2]	0.16
Gait velocity (km/h)	**0.44 [0.26; 1.80]**	**0.80 [0.68; 2.0]**	**0.046**

## Discussion

To better understand the gait analysis used in this study, let us imagine how a man moves in the surrounding space.

Human walking is driven by a complex interaction between the nervous and musculoskeletal systems. If the corticospinal pathways of the spinal cord are damaged due to a trauma or vascular catastrophe, impulse conduction to the peripheral motor neuron is impaired. While maintaining the ability to walk, this individual develops a pathological gait pattern. Like normal walking, abnormal walking consists of several stages. The period of a double step (a full walking cycle) is the time between two consecutive contacts of the supporting leg heel with the ground. The hip joint is bent, the knee is straightened, and the ankle joint is in the dorsiflexion position. The entire period of the double-step consists of the stand phase and the swing phase [11, 12].

**Figure 3.**
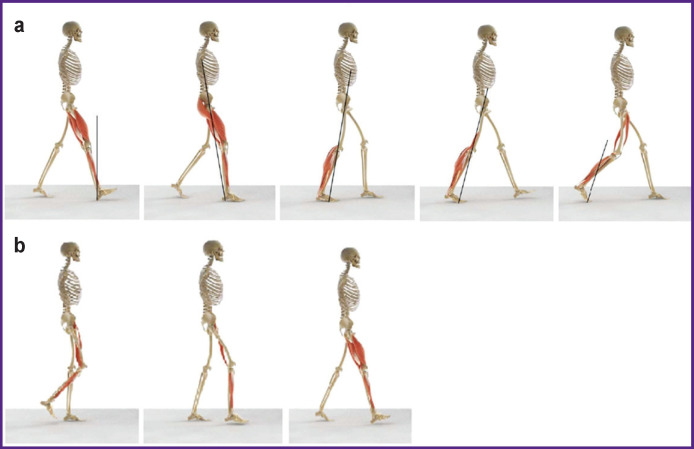
The main phases of the double-step period: (a) stand phase; (b) swing phase

In the stand phase (Figure 3 (a)), the body weight falls on the entire supporting foot. The body’s spatial movement is driven mainly by inertia, with minimal muscle activity. The leg is bent in the hip joint, slightly flexed in the knee joint, and the ankle joint is in the middle position between the dorsal and plantar foot flexion. At the end of the stand phase, the body is pushed forward and upward, mainly due to activation of the ankle and foot muscles. In contrast to the early stand phase, the body movement occurs mainly due to muscular strength [11, 12].

In the swing phase (Figure 3 (b)), the foot is lifted from the surface. The body movement is performed due to inertia. The leg at the hip and knee joints is bent as much as possible. The swing phase, like the entire walking cycle, ends with the contact between the foot and the support [11, 12].

In the present study, objective indicators of gait in spastic tetraparesis were obtained. Specifically, there was an increase in the stand phase (from 81.9 [76.1; 89.2] to 85.3 [74.4; 90.2]%; p=0.97) with a shortening of the swing phase and the period of the double step (from 0.50 [0.45; 0.96] to 0.40 [0.34; 0.66]; p=0.4); relatively to the target values, the changes were 60% — for the stand phase and 1.41 — for the double-step period. Numerical analysis of the hip, knee, and ankle joints movement showed a decrease in the range of motion in the hip joints with an increase of this range in the knee and ankle ones. Thus, the subjective definition of “paretic gait” can be supplemented with numerical values. This allows us to express the severity of the disorder and its changes during treatment.

Four patients with central spastic tetraparesis who underwent gait assessment before and after treatment showed an improvement according to several parameters. Given the pilot nature of the study and the small sample size, this work cannot answer the question, which of the treatment methods is more effective in improving the patient’s gait; furthermore, the results do not allow us to conclude about the universality of the proposed parameters. Yet, the demonstrated 2DGA recording of the patient’s gait and its changes during treatment open the way for further research in this area.

Comparative evaluation of data from sensors attached to the hip, knee, and ankle joints did not reveal significant differences between the left and right lower limbs (p>0.05). We, therefore, conclude that patients with central spastic tetraparesis have similar hypertonicities on the left and right sides. This result is probably due to the “symmetry” of paresis in this group of patients, which is usually observed with this type of spinal cord lesion. In patients with a lesion at a different topical level, changes in muscle tone can be entirely different. The present results imply that gait abnormalities in patients with cervical spinal cord lesions below the C_2_ vertebra can be diagnosed by using a video analysis of one leg only.

Our analysis of gait changes after the treatment showed a statistically significant decrease in the duration of the stand phase (p=0.035) and an increase in the swing phase (p=0.035). There was also an increase in the range of motion in the hip joints (p=0.011) and the gait velocity (p=0.046). There was a decrease in the “waiting” time before the walking began and an increase in the flexion amplitude in the hip joints.

The results of the study show that 2DGA can be applied to the assessment of gait disorders in patients with spastic tetraparesis of various etiologies. In the future, we are going to study whether there is a correlation between the walking stereotype abnormalities identified by video analysis, with a decrease in quality of life, everyday activities, and mobility of patients.

## Conclusion

2D gait analysis is a promising method for quantifying gait disturbances in patients with spastic tetraparesis. It allows one to identify characteristic gait patterns in this group of patients, including an increase in the stand phase with a shortening of the swing phase and the double step period, as well as changes in the range of motion in the hip, knee, and ankle joints. Further use of 2D gait analysis will focus on evaluating the effectiveness of treatment in such patients.

## References

[1] Janssen I., Nouri A., Tessitore E., Meyer B. (2020). Cervical myelopathy in patients suffering from rheumatoid arthritis — a case series of 9 patients and a review of the literature.. J Clin Med.

[2] Ponomarev G.V., Skoromets A.A., Krasnov V.S., Rodionova O.V., Glistenkova D.D., Porkhun N.F., Dambinova S.A. (2018). Vascular myelopathy: causes and mechanisms, possibilities of diagnosis and treatment.. Nevrologia, nejropsihiatria, psihosomatika.

[3] Natale M., Mirone G., Rotondo M., Moraci A. (2012). Intrathecal baclofen therapy for severe spasticity: analysis on a series of 112 consecutive patients and future prospectives.. Clin Neurol Neurosurg.

[4] Fjelstad A.B., Hommelstad J., Sorteberg A. (2009). Infections related to intrathecal baclofen therapy in children and adults: frequency and risk factors.. J Neurosurg Pediatr.

[5] Pittelkow T.P., Bendel M.A., Lueders D.R., Beck L.A., Pingree M.J., Hoelzer B.C. (2018). Quantifying the change of spasticity after intrathecal baclofen administration: a descriptive retrospective analysis.. Clin Neurol Neurosurg.

[6] Biering-Sørensen F., Nielsen J., Klinge K. (2006). Spasticity-assessment: a review.. Spinal Cord.

[7] Ashworth B. (1964). Preliminary trial of carisoprodol in multiple sclerosis.. Practitioner.

[8] Suponeva N.A., Yusupova D.G., Zhirova E.S., Melchenko D.A., Taratukhina A.S., Butkovskaya А.A., Ilyina K.A., Zaitsev A.B., Zimin A.A., Klochkov A.S., Lyukmanov R.Kh., Kalinkina M.E., Piradov M.А., Kotov-Smolensky A.M., Khizhnikova A.E. (2018). Validation of The Modified Rankin Scale in Russia.. Nevrologia, nejropsihiatria, psihosomatika.

[9] Langerak N.G., Lamberts R.P., Fieggen A.G., Peter J.C., Merwe L., Peacock W.J., Vaughan C.L. (2008). A prospective gait analysis study in patients with diplegic cerebral palsy 20 years after selective dorsal rhizotomy.. J Neurosurg Pediatr.

[10] Borzikov V.V., Rukina N.N., Vorobyova O.V., Kuznetsov A.N., Belova A.N. (2015). Human motion video analysis in clinical practice (review).. Sovremennye tehnologii v medicine.

[11] Morozov I.N. (2011). Pozvonochno-spinnomozgovaya travma: vosstanovitel’noe lechenie v promezhutochnom i pozdnem periodakh..

[12] Vitenzon A.S., Petrushanskaya K.A., Skvortsov D.V. (2005). Rukovodstvo po primeneniyu metoda iskusstvennoy korrektsii khod’by i ritmicheskikh dvizheniy posredstvom programmiruemoy elektrostimulyatsii myshts.

